# Age-Friendly University Initiative Applied to Health Professions Education Programs

**DOI:** 10.1093/geroni/igaa057.1793

**Published:** 2020-12-16

**Authors:** Marilyn Gugliucci

**Affiliations:** University of New England College of Osteopathic Medicine, Biddeford, Maine, United States

## Abstract

Opportunities for health professions (HP) education programs to make a mark in the Age Friendly University (AFU) initiative abound. Key approaches are introduced, and insights for mounting these efforts are discussed, for HP education programs based on the AFU global network initiative and the Academy for Gerontology in Higher Education (AGHE). Higher Education Institutions (HEIs) that offer HP education, have various options to establish and enhance gerontology/geriatrics competence and confidence for their students. AFU guiding principles applicable to HP education allow health gerontology faculty to be catalysts to promote and integrate the AFU guiding principles within their program’s existing curriculum. This can contribute to their institution’s readiness to apply for the AFU designation, advance the institution’s age friendliness, and/or set up the program to apply for the AGHE Program of Merit (POM) for Health Professions Programs. All prepare future HP professions providers to improve older adult health care. Part of a symposium sponsored by Directors of Aging Centers Interest Group.

